# A framework for elderly participation in Primary Health Care in Tabriz Health complexes

**DOI:** 10.1186/s12877-023-04217-1

**Published:** 2023-08-21

**Authors:** Mahdieh Najafi, Kamal Gholipour, Mohammad Amerzadeh, Mohammad Zakaria Kiaei, Rohollah Kalhor

**Affiliations:** 1 Student Research Committee, School of Public Health, Qazvin University of Medical Sciences, Qazvin, Iran; 2 Tabriz Health Services Management Research Center, Department of Health Policy and Management, School of Management and Medical Informatics, Tabriz University of Medical Sciences, Tabriz, Iran; 3 Social Determinants of Health Research Center, Research Institute for Prevention of Non-Communicable Diseases, Qazvin University of Medical Sciences, Qazvin, Iran; 4 Health Services Management, School of Public Health, Qazvin University of Medical Sciences, Qazvin, Iran; 5 Social Determinants of Health Research Center, Research Institute for Prevention of Non-Communicable Diseases, Qazvin University of Medical Sciences, Qazvin, Iran

**Keywords:** Elderly, Primary Health Care, Health complexes

## Abstract

**Background:**

A framework for increasing elderly participation in Primary Health Care (PHC) is a vital issue considering the growing population. After examining the situation and elderly participation in the provision of PHC in the health complexes of Tabriz City, the present study presents the framework of elderly participation in PHC.

**Methods:**

This is a mixed-method study. First, we reviewed the models of elderly participation in PHC worldwide using a comprehensive search of literature. Then, we extracted the service providers’ and the elderly's views regarding the obstacles and solutions for the elderly participation in PHC in Iran using the interviews and focus group discussions (FGD). We conducted three FGDs (8–10 people) and seven individual interviews. Data were analyzed using the content analysis method. We developed the proposed framework for the participation of the elderly in PHC using a panel of experts and checked and confirmed the framework's validity using the Delphi technique with 11 experts from the content validity index and modified kappa coefficient.

**Results:**

Based on the result of included studies in the systematic review, the characteristics of the participation models were classified into five areas: the characteristics of the service user, the main facilitator of the intervention, the type of ownership of the center, the subject and the method of participation. The solutions and obstacles, and problems presented by the service providers and users in different areas include 12 themes (elderly participation, home care, and self-care, respect for the elderly, cooperation of different organizations, service package for the elderly, referral system, planning for the elderly, considering insurance for the elderly, the role of informing the elderly, mental health of the elderly, physical space of centers and training of elderly caregivers) and 46 sub-themes. The final framework also includes five themes (approaches and strategies to attract participation, indicators, and consequences of participation of the elderly, implementation strategies of elderly care, implementation infrastructure and goals and areas of participation of the elderly) and sub-themes.

**Conclusion:**

The results of the study indicate that the final framework obtained should be used based on a systematic model for elderly participation in PHC and should be implemented and followed up based on local strategies and specific indicators, considering all capacities.

## Background

Improvement in living conditions and increase in life expectancy have ended up in the ageing phenomenon in societies. The increase in the elderly population is one of the most critical economic, social, and health challenges in the twenty-first century [1]. Today, about 700 million people worldwide are over 60 years old [2].

With the increase in the elderly population, their health problems have also increased. Chronic diseases are the most common causes of disability in the elderly, so in recent years, due to the increase in society's age, the frequency of this disease and its symptoms have become more noticeable, and it has been given a higher priority [3, 4]. Patients suffering from chronic diseases suffer from various problems that reduce their quality of life due to their painful and debilitating nature. Various studies have found that this debilitating nature leads to a decrease in the quality of life in these patients. Medical, psychological, and moral attention to this age group is necessary [5].

Many efforts are made to improve the quality and quantity of life for society’s elderly. Studies show that about 60% of healthcare costs are related to elderly people [6]. One of the most critical issues in improving the lives of the elderly is helping them to increase their participation in Primary Health Care (PHC), which can be an essential help for this group along with ageing. Studies show that PHC can cover 75–85% of elderly needs and specialist consultation is only necessary 10–12% of the time [7]. The elderly may receive services from various providers, in different settings, often poorly structured [8]. Promoting the participation of elderly patients may improve the quality of care and lead to better adherence to prescribed medications and recommendations. It also causes high satisfaction with care and improvement of health status. To receiving better services in the elderly, using PHC institutions are highlighted [9, 10]. Considering the importance of the elderly participation in PHC, we studied elderly participation status in PHC in Tabriz city to provide a framework for improving the elderly participation in PHC.

## Methods

### Study design

This is a mixed quantitative and qualitative study method, which first examined the literature on the models of elderly participation in PHC using a comprehensive review and systematic search of studies and articles related to elderly participation.

### Sampling and recruitment methods

We searched English and Farsi sources in the relevant databases, including PubMed, Web of Science, Science Direct, Scopus, SID, Iran doc, and Magiran, using keywords (partnership, elderly, ageing, primary health care). Then, we conducted interviews and focus group discussions (FGDs). We investigated service providers' views regarding obstacles and solutions at this stage using individual interviews, FGDs, and semi-structured guide. We used qualitative and phenomenological research methods in the second and third stages. The primary goal of the phenomenological method is to understand the main structure of human experienced phenomena and to achieve the conceptual depth of lived experiences.

### Data collection

In total, we interviewed seven people from the target group. The tool used to collect data was an interview guide. We conducted a semi-structured face-to-face interview. The interviews were conducted by one of the research group members until saturation. The duration of each interview was 60 min on average.

The interview guide was in 5 main topics, including the implementation strategies of elderly care, implementation infrastructure, approaches and strategies to attract participation, goals, and participation areas, and indicators and consequences.

The FGDs enabled us to collect rich information through the interaction between the participants. The semi-structured form of questions in the FGDs resulted in extensive, rich information.

We conducted three FGDs (8–10 people) with elderly people and seven interviews with service providers. The duration of the FGD was 90 min and 60 min for interviews. We recorded the FGDs and interview via written consent and transcribed it verbatim. At the end of each session, the data was analyzed manually using the content analysis method. We used purposive sampling method to conduct interviews and FGDs. If some people who were eligible to contribute to participate in the study were not able to participate in the FGDs, they were arranged to conduct an interview, and their experiences were also reviewed in this way. There are no differences between participants in interviews and FGDs in accordance with the inclusion criteria.

Participants were permitted to leave the research at any stage. The participants permitted us to record the meetings using voice-recorder, and at least two researchers facilitated each FGD, one interviewer and one note taker. After the meeting, we read the contents of the notes to the attendees and asked them to confirm. Then we extracted the codes, concepts and content to categorize the concepts. Before the FGD, we provided the experts with a summary of the findings of the first research aim.

**Fig. 1:**
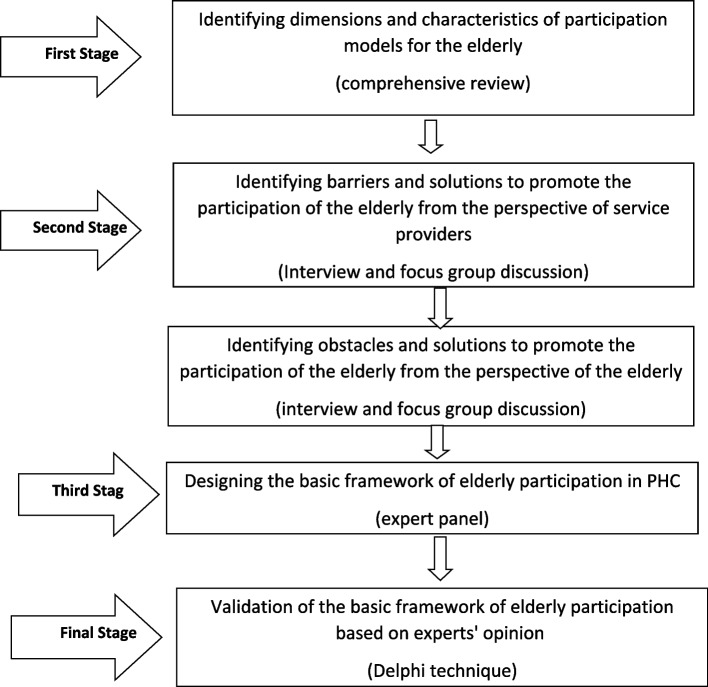
Flowchart of study design and methods

Finally, using the expert panel, we finalized the dimensions and components of the framework for elderly participation in PHC care based on the expert panel opinions and obtained the initial framework. At this stage, we classified the findings of the previous stages (comprehensive review, qualitative study at the level of organizational units and qualitative study at the policy level) and presented them as a final table. Subsequently, we used professors, experts, and expert panel opinions to validate the framework. Figure 1 shows the different stages:

Inclusion Criteria for Elderly people:

- Being older than 65 years
- Elderly people referring to PHC centers

Inclusion Criteria for service providers:

- Service providers and experts who take care of the elderly people
- Service providers and experts who play managerial role in elderly care programs
- More than 5 year experiences in elderly care programs

Exclusion criteria:

- Elderly people who were not physically able to sit in FGD
- Participants who were not willing to participate in the study.

## Data analysis

We used the thematic analysis method to analyze data and extract models of elderly participation in PHC from the articles obtained from the comprehensive review and classification of the contents of the articles included in the study. For this purpose, we analyzed the models identified in the literature review in data and program structure, the process of care and participation, the consequences of care and participation, and the role of care providers and the elderly people. We conducted data analysis manually.

We analyzed the data collected from the FGD and interviews using the content analysis method. The data analysis started from the first group discussion session in parallel with the subsequent sessions (simultaneous analysis). We read the notes several times to obtain a general understanding of the text. We extracted the solutions and obstacles to the elderly participation in PHC from the interviewees’ opinions in the meeting. Then we identified themes and sub-themes. We used a content validity index and modified kappa coefficient in a questionnaire to confirm the framework’s validity after developing the proposed framework using the Delphi technique with 11 experts. We calculated the probability of chance agreement to calculate kappa coefficient. For this purpose, the binomial random variable formula was used:

$$\mathrm{Pc}=\left[\frac{\mathrm{N}}{A!\left(N-A\right)!}\right].{5}^{N}$$

N: number of experts.

A: The number of experts who marked items 3 and 4 (the number of those who agreed).

Then, in the next step, kappa coefficient (k*) was calculated using the ratio of agreement on the relationship (or I-CVI) and the probability of chance agreement as follows:

$${K}^{*}=\frac{I-CVI-{P}_{c}}{1-{P}_{c}}$$

Then Polit et al. calculated and suggested fair, good and excellent values for the calculation of I-CVI, as well as k* (kappa coefficient above 0.40 is fair, between 0.60 and 0.74 is good and above 0.74 is excellent). We used the values of this table to judge the experts’ opinions and measured the framework's validity in the experts’ opinions. We examined twelve items: the implementation capability of the framework, its compliance with the upstream policies, the acceptability by the stakeholders, the efficiency, the flexibility (ability to adapt to the conditions), the effectiveness, simplicity (comprehensibility), coherence and integration between the framework components, comprehensiveness sequence of components, proportionality and balance between the framework components and how suitable the proposed framework is for Iran’s health system. Experts expressed their opinions on a Likert scale in four options: very low, low, high, and very high. Items 1 and 2 were considered being against, and items 3 and 4 were considered agreeing, and we obtained I-CVI and k* in this way [11].

### Ethical considerations

The ethical issues observed in this study are: obtaining informed consent from the participants verbally before entering the research, receiving a written letter from the university to make the necessary arrangements, registering the proposal and registering the code of ethics in the University of Medical Sciences, coordination of place and time with participants in the project, coding the names of the participants before conducting the discussion to ensure the confidentiality of all information obtained from the participants in the research and not mentioning the names of the participants, obtaining consent to record group discussions and interviews and non-recording in cases where consent has not been obtained, viewing the information obtained from the FGD by the participants for confirmation.

The current study, with the registration code (IR.QUMS.REC.1397086) aims to investigate the status of participation of the elderly people in primary health care in the health complexes of Tabriz city and provide a framework for participation based on the results obtained.

## Results

Figure 1 shows the method. The main participatory methods obtained in this extensive review study by examining many articles included seven methods, respectively: team programs, integrated care, counselling method, interview and audio recorder, leaflet, home, workshop and class. The most used method was an interview and audio recording, used in the 12 final studies. The method used most, along with other participation methods, was the workshop and class, which were used more than others in five studies.

The fields of participation obtained from the review of these articles included chronic diseases, mental diseases, social diseases, PHC, polypharmacy patients, and self-care. Most of the studies conducted on mental diseases were used in 11 studies. The lowest was in polypharmacy patients, used in only two studies.

Most of the studies were conducted in government centers; 24 of them and only five were conducted in the private sector. Most researchers have also attracted government support in conducting their studies.

Most providers in the interventions obtained from the studies (general practitioner, specialist, health coach, research assistant and nurse) were nurses, used as a guide in 12 studies. Also, this option was the most service provider that, with other service providers (in 6 cases out of 12 cases), provided services to the elderly people during the interventions.

**Fig. 2:**
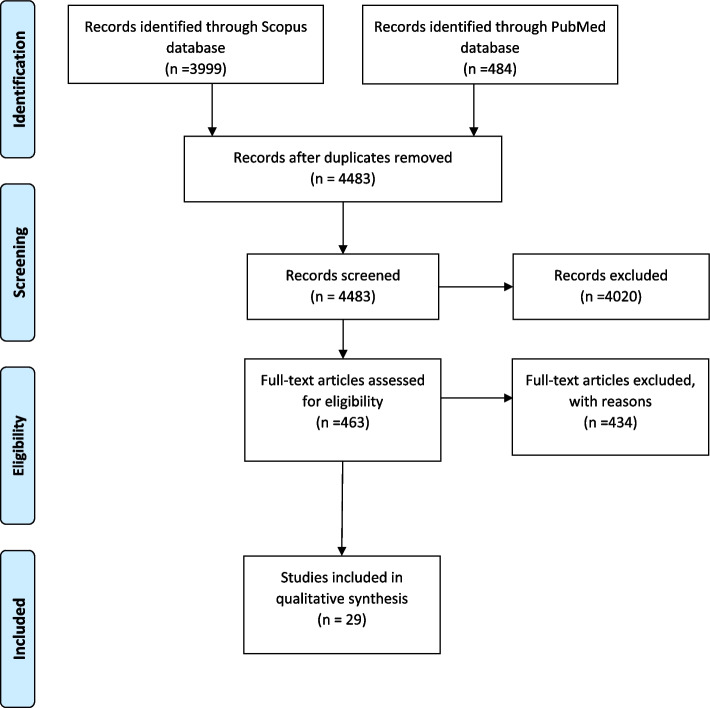
PRISMA flow diagram search and article selection process

In most of the studies, the recipient of the intervention was only the elderly people in the 29 studies obtained; therefore, in the options that the service provider should also be included in the training, in 6 cases of the study, it is in a limited number. Figure 2 shows the PRISMA flow diagram search and article selection process. We used findings from comprehensive review to design our interview guide. It helped us to get familiar with the literature of the elderly (Table 1).

**Table 1:** The Results of the Comprehensive Review Regarding Goals, Methods, and Focus Areas of Elderly Participation Frameworks in Primary Health Care

Title	Participation Method							Intervention Goal		Center		Participation topic					
	**Team programs**	**Integrated care**	**Counselling method**	**Leaflet**	**At home**	**Workshop and class**	**Interview and audio recorder ا**	**Provider**	**The Elderly**	**Private**	**Public**	**Self-care**	**Polypharmacy patients**	**PHC**	**Social diseases**	**Mental diseases **	**Chronic diseases**
1Daaleman, T. P, et al. [12]							*		*		*						*
2- Koop’s van’t Jag, R, et al. [13]							*		*		*			*			
3Aschbrenner, K. A, et al. [14]							*		*		*					*	*
4- Michelle Howard, et al. [15]							*		*		*						*
5- De Jong, C. C, et al. [16]							*		*		*	*			*	*	
6- Kastner, M, et al. [17]							*		*		*						*
7- Walters, K, et al. [15–18]							*		*	*		*			*		
8-Raymond Wetzel’s, et al. [16–19]							*		*		*			*			
9- Lopez Mateo’s, et al. [17–20]						*	*		*		*						*
10- Arlene Fink, et al. [21]						*	*		*		*	*		*			
11- Gum, A. M, et al. [19–22]	*					*	*		*		*					*	
12- Olsson, I. N, et al. [23]					*		*	*	*		*		*				
13-Aileen WK Chan, et al. [21–24]						*		*	*	*					*		
14- Lousier, M. T, et al. [25]						*			*		*						*
15- Bartels, S. J, et al. [26]	*					*			*		*					*	*
16- Fuller, J, et al. [27]		*				*		*	*		*					*	
17- Wetherill, J. L, et al. [25–28]						*			*		*					*	
18- Tiedemann, A, et al. [26–29]					*	*			*	*		*					
19-Tina Aaen Geest, et al. [27–30]				*		*		*	*		*	*					
20- Moral RR, et al. [31]			*			*		*	*		*		*				*
21- Van der Weele, G. M, et al. [32]			*		*				*		*					*	
22- Pfaff, J. J, et al. [33]					*				*	*		*				*	
23- Harris, T, et al. [34]			*						*		*	*					
24- Vedel, I, et al. [35]		*						*	*		*			*			
25. Horne et al. [36]			*		*				*			*					
26- Callahan, C. M, et al. [34–37]	*								*		*					*	
27- Billington, J. [38]	*								*	*						*	
28 Everett, C. M, et al. [39]	*								*		*						*
29- Taina Rantanen, et al. [37–40]	*								*		*	*					

* indicates the report of the subject in the study

As mentioned, we used the interviews and FGDs to prioritize the existing themes regarding the obstacles, challenges and solutions faced by the elderly in providing and receiving services from specialists, officials and environmental health experts in health centers. The final themes are in Tables 2 and 3.

**Table 2:** Obstacles and challenges presented by recipients and service providers by areas

Theme	Sub-theme	Quote
**Participation of the elderly**	Weakness in establishing proper communication between caregivers and the elderly	*"Most colleagues have problems in communicating with the elderly and they are not experts in how to talk, how to deal with them "*
	Reduced ability of the elderly to learn	*"The elderly may not be learning."*
**Care and self-care**	Inadequate participation of caregivers in the self-care of the elderly	*" We have a package for self-care for the elderly. It is very good, provided that caregivers are interested."*
**Respect for the elderly**	Defects in respect for the elderly	*"The elderly receive services, but if it is done in a proper way and with respect, it will be great."*
**Cooperation of different organizations**	Weak cooperation between different organizations regarding the elderly service	*" Non-cooperation of different organizations in caring for the elderly is a big problem that our society is facing, while different organizations should participate in this matter."*
**The service package for the elderly**	The incompleteness of the service package for the elderly	*" Our package is incomplete and it is becoming more and more limited."*
**Referral system**	Caregivers' indifference to the referral of the elderly to health centers	*" Due to lack of awareness, the elderly does not visit the centers regularly, and the caregivers do not follow up appropriately"*
	Inadequate work of health care workers in filing health records for the covered population	*" Information about the citizens' health case is weak. My friend and I went on our own without being informed."*
	Insufficient cooperation of the elderly in visiting health centers	*"Some elderlies do not do much in visiting doctors and health centers, and health care providers are obliged to identify these people and train them to follow up on their health status."*
	Inappropriate environment, conditions and facilities of health centers for the elderly	*"One weaknesses of health centers is that all services are not available in a health complex, and the elderly have to go to different centers in the city to get different services."*
	Provision of incomplete services to the elderly	*" The services that are necessary for the elderly should consider a wide range. Mental health services, prevention, care, education, and other services should be considered except services for the elderly. "*
**Attention to insurance for the elderly**	Inadequate attention to welfare for the elderly	*"The next challenge was the lack of insurance support for medicine and treatment costs for the elderly."*
	Lack of financial ability of the elderly to refer to health centers	*"The elderly cannot really afford the high costs of treatment"*
	Insufficient attention of insurance to the elderly	*" Non-coverage of old-age expenses by insurance and lack of attention of insurances to the elderly have raised the problems."*
**Role of informing the elderly**	Insufficient use of the elderly as health volunteers	*"They can use more of the elderly as health volunteers, but there is no specific process for it."*
**The elderly’s mental health**	Insufficient attention of the families to the elderly	*"Now that we are old, no one remembers us anymore."*
**The physical space of the centers**	Inappropriate physical space of the health centers for the elderly	*"At least 30% of our centers, their physical condition is not favorable"*
**Training of elderly caregivers**	Insufficient skills of doctors and caregivers in caring for the elderly	*"We have never studied the elderly, we have not studied anything about psychology, there are some things that are out of our scope"*

**Table 3:** The solutions provided by recipients and providers by themes

Theme	Sub-theme
**Participation of the elderly**	Participation of the elderly in providing services and self-care
	Improving communication with the elderly
	Promoting care for the elderly
**Home care and self-care**	Self-care empowerment of the elderly
	Providing home care services
**Respecting the elderly**	Creating a culture of caring for the elderly
	Improving the mental condition of the elderly
**Cooperation with various organizations,**	Improving inter-sectoral cooperation
	Strengthening the services and facilities of health centers
	Creating recreational facilities for the elderly
**Service Package for the elderly**	Expanding the service package for the elderly
	Increasing the access of the elderly to services
**The referral system**	Strengthening the referral system in the care of the elderly
	Informing about services for the elderly
**Planning for the elderly**	Planning to improve access to services for the elderly
	Expanding elderly-friendly centers
	Strengthening prevention services for the elderly
	Informing the elderly of their rights in society
**Paying attention to insurance for the elderly,**	Expanding coverage Insurance services for the elderly
**The role of informing the elderly**	Implementing and controlling the role of educating and informing the elderly
	Holding recreational programs for the elderly
	Providing drug counseling services to the elderly
**Mental health of the elderly**	Strengthening mental health services for the elderly
	Strengthening the role of the elderly in the family and society
	The important role of elderly-friendly centers
	Expanding the social activities of the elderly
**The physical space of the centers**	Improving the elderly friendly centers and their physical space,
**Training elderly caregivers**	The importance of training caregivers

**Fig. 3:**
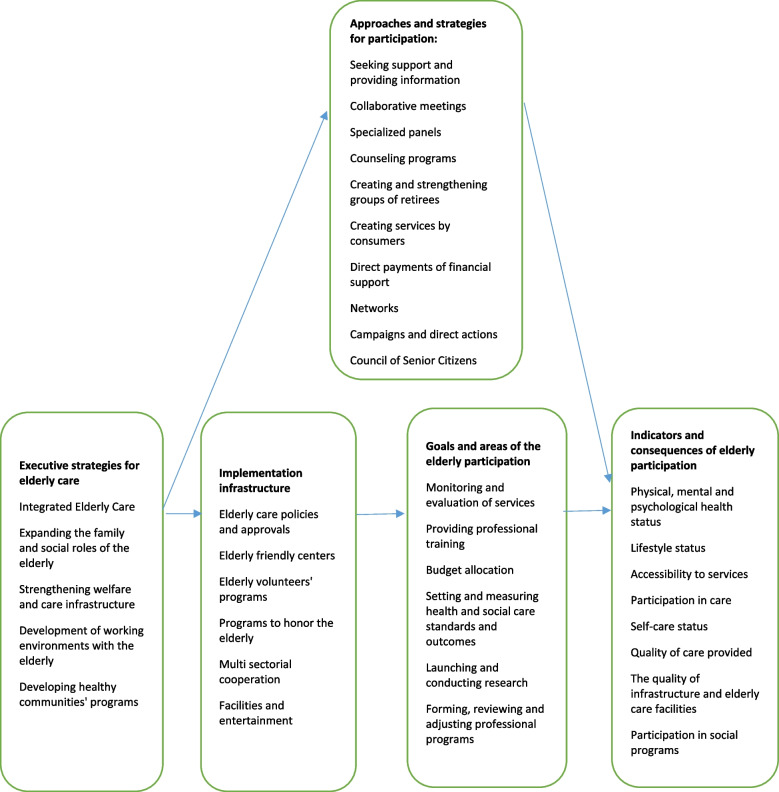
Final framework for the elderly participation in PHC

The results obtained from the comprehensive review of texts, interviews and FGDs were presented to the expert panel. In the results of this group’s comments, discussions and debates, the final framework was obtained in Fig. 3.

The designed model includes five main areas (executive strategies for elderly care, implementation infrastructure, approaches and strategies for participation, goals, and areas of the elderly participation, and indicators and consequences of elderly participation).

Executive strategies show the system's point of view regarding the issue of attracting elderly participation. This area requires the activity of the upper authorities in the direction that the fields should be provided by these people first.

For implementing the system's perspective on the elderly, there is a need for a series of requirements and infrastructures for elderly participation. In the final model, we mentioned six executive infrastructures.

We need to use ten approaches and strategies to attract participation in the model.

By preparing the necessary infrastructure and using participation approaches, we can improve the elderly participants in the sub-fields of monitoring and evaluating services, providing professional training, allocating and determining budgets, determining and measuring standards, etc. Finally, the elderly participation and the measurement of eight primary outcome indicators are considered to determine the success percentage in the model.

As seen in the table below, the experts evaluated the feasibility and implementation, flexibility (ability to adapt to the conditions) simplicity and comprehensibility of the proposed framework (k* = 0.59–0.40).). Besides, the stakeholders evaluated the acceptability of the proposed framework (k* = 0.74–0.60).

The compliance of the proposed framework with upstream policies, its efficiency and effectiveness, the coherence and integration, the sequence of the components, and the appropriateness and balance between the components were evaluated as excellent (k* > 0.74). In general, the experts evaluated the proposed model as excellent and suitable for Iran's health system (k* = 0.74–0.60) as shown in Table 4.

**Table 4:** The results of experts' opinions and the coefficient of agreement between them in each theme

Row	**Area**	**I-CVI**	**K**	**Status of the area**
1	Implementation capability of the proposed framework	0.54	0.42	Acceptable
2	Compatibility of the proposed framework with upstream policies	0.84	0.85	Excellent
3	The accessibility of the proposed framework by the stakeholders	0.69	0.66	Good
4	Efficiency of the proposed framework	0.85	0.84	Excellent
5	Flexibility (ability to adapt to conditions) of the proposed framework	0.54	0.42	Acceptable
6	Effectiveness of the proposed framework	0.92	0.92	Excellent
7	Simplicity (comprehensibility) of the proposed framework	0.62	0.54	Acceptable
8	Coherence and integration between the components of the proposed framework	0.77	0.76	Excellent
9	The comprehensiveness of the proposed framework	0.85	0.84	Excellent
10	The sequence of the components of the proposed framework	0.85	0.84	Excellent
11	Proportion and balance between the components of the proposed framework	0.92	0.92	Excellent
12	Overall, how appropriate do you consider the proposed framework for Iran's health system?	0.85	0.84	Excellent

## Discussion

This study investigated the elderly participation status in PHC in Tabriz City to provide a framework for improving elderly participation in PHC. The final framework includes five themes and sub-themes.

During the interviews and FGDs, we categorized the solutions presented by the service providers and receivers in different themes, including 12 themes. We also categorized the obstacles and problems presented by the service providers and recipients by themes, including 12 themes.

The final model includes five themes (approaches and strategies to attract participation, indicators and consequences of the elderly participation, organizational strategies of elderly care, executive infrastructure and goals and areas of the elderly participation) and sub-branches.Another study found three themes as facilitators of the elderly participation in PHC: person and family-focused care, self-management resources, and successful collaborative practice [41]. In line with our findings, A study by Wetzel et al. showed that most general practitioners had a positive view of the participation of elderly patients. Nevertheless, the main obstacle for physicians was the lack of time. It is difficult to increase their participation, and it will be accessible when general practitioners reach a broader concept and are supported by various motives [42, 43].

In this study, the barriers to elderly participation, the cooperation of various organizations, the service package for the elderly and the training of elderly caregivers were in line with Molzadeh’s findings. In both studies, the reasons for the low level of participation of the elderly in health education programs include the inability to travel, the lack of transportation facilities, the weather, the long distance of villages from the health center, repetitive and prolonged educational topics, the busyness of the family in accompanying the elderly, the lack of information about the dates of the programs and forgetfulness. In the present study, the problems mentioned above are sub-themes of cooperation of different organizations, the service package for the elderly and the training of elderly caregivers, along with other specific and general causes in the service delivery system, in reducing the participation of the elderly in educational programs. It will encourage those involved in this field to evaluate educational services more comprehensively and formulate appropriate and practical solutions [44, 45]. A similar study found several reasons for the lack of elderly participation in PHC, including poor communication among service providers and patients, a complicated health care system, barriers in information system, a lack of consistency of service delivery, and inappropriate follow-up system [41]. In line with our findings a study found that the elderly were not aware of the available resources [46].

Wetzel et al., indicates that informing about health care usage, giving information to choose a care provider, and facilitate contact the service provider (providing patient information, preparing for active participation) improved the elderly participation because people had acquired much information about it [47]. Other studies revealed that access to PHC was higher in people with chronic disease showing that healthcare providers prioritize them with higher needs [48, 49]. These results are consistent with the present study regarding the areas obtained in the elderly participation solutions.

According to one study, the views of the elderly were divided into four major categories: doctor-patient interaction, issues related to general practitioners, patient-related issues, and contextual factors. It found that people over 70 wanted to participate in their care. However, their definition of participation was focused more on “care relationships” and “receiving information” rather than “active participation in decision-making” [50]. Similarly, in our study, among the solutions provided by the service recipients, doctor-patient interaction in the service package for the elderly, issues related to GP in the education of elderly caregivers and issues related to the patient self-care of the elderly can be seen. However, in Bastian's et al.’s study, the elderly’s definition of participation is more focused on “care relationships” and “receiving information” than on “active participation in decision-making”. In contrast, in our study, the elderly emphasized active participation in their decisions. Generating consistency via standardization through health information technology can improve PHC system and healthcare delivery [51]. Electronic health records play significant role in integrating PHC and care management. They can facilitate the flow of necessary information among different providers, which needs specific attention in Iran [52]. A systematic review found that electronic information systems can improve patient safety, decrease medical errors, enhance access to results and data, expand referral processes and help PHC system work more efficiently [53].

The results of the present study indicate that self-care and training are the themes. Despite the interest of the elderly in self-care, there is not enough training in this field. This finding is in line with Salimi et al. results indicating that if the elderly has correct information about a healthy lifestyle, many of their problems can be prevented or controlled well and planned to improve their quality of life.

According to the WHO report about healthcare systems for an ageing population published in 2014, the main areas to improve elderly participation include: supporting the elderly’ independence, helping the elderly to live well with long-term or stable conditions, providing quick support and close to the nursing home, planning for the elderly, providing rehabilitation care for the elderly, training nursing care for the elderly and provision of integrated care for the elderly. They are in line with the themes of our proposed framework: implementation strategies for elderly care, implementation infrastructure, approaches and strategies to attract participation, goals and the areas of elderly participation and the indicators and consequences of elderly participation.

### Limitations

Like many other studies, the present study faced limitations, including the fact that experiences of only Tabriz as a metropolitan city was investigated. Therefore, generalization of the results to other places may not be reasonable. It is suggested that elderly participation in primary care be studied on a larger scale in the future. Another limitation of the present study is the examination of the issue only from the point of view of experts and the elder people, and the practical limitations of the participation of the elder people in primary health care have not been investigated.

## Conclusion

Based on the findings of the study, the problems caused by elderly participation include weak elderly participation, incomplete home care and self-care, incomplete respect for the elderly, incomplete cooperation of various organizations, incomplete service package for the elderly, weakness in the referral system, insufficient attention of the insurance to the elderly, the weakness in the informing the elderly, inappropriate treatment of the elderly’s mental health, inappropriate physical space of the centers and inadequate training of the elderly caregivers. The solutions include improving the elderly participation, home care and self-care, respect for the elderly, cooperation of various organizations, service packages for the elderly, referral system, planning for the elderly, attention to insurance for the elderly, informing the elderly, the elderly’s mental health, the physical space of centers and the training of elderly caregivers. Finally, the health system should use an appropriate framework to attract elderly participation. Therefore, the infrastructures that exist in various organizations should be used as much as possible, and this work should be carried forward with the help of participation approaches. The progress of participation of the elderly should be monitored by determining a series of indicators.

## Data Availability

The datasets used and/or analyzed during the current study available from the corresponding author on reasonable request. The entire dataset is in Farsi language. The Data can be available in English language for the readers and make available from the corresponding author on reasonable request.
